# The Cholinergic Drug Pyridostigmine Alleviates Inflammation During LPS-Induced Acute Respiratory Distress Syndrome

**DOI:** 10.3389/fphar.2021.624895

**Published:** 2021-05-04

**Authors:** Pamela Nithzi Bricher Choque, Rodolfo P. Vieira, Luis Ulloa, Caren Grabulosa, Maria Claudia Irigoyen, Katia De Angelis, Ana Paula Ligeiro De Oliveira, Kevin J. Tracey, Valentin A. Pavlov, Fernanda Marciano Consolim-Colombo

**Affiliations:** ^1^ Laboratory of Pulmonary Immunology, Postgraduate Program in Medicine, Universidade Nove de Julho (UNINOVE), São Paulo, Brazil; ^2^ Post-graduation Program in Bioengineering and in Biomedical Engineering, Universidade Brasil, São Paulo, Brazil; ^3^ Brazilian Institute of Teaching and Research in Pulmonary and Exercise Immunology (IBEPIPE), São Paulo, Brazil; ^4^ Federal University of São Paulo (UNIFESP), Post-graduation Program in Sciences of Human Movement and Rehabilitation, São Paulo, Brazil; ^5^ Departament of Physiology, Federal University of São Paulo (UNIFESP), São Paulo, Brazil; ^6^ Department of Anesthesiology, Duke University Medical Center, Durham, NC, United States; ^7^ Hypertension Unit, Heart Institute (INCOR), Medical School of University of São Paulo, São Paulo, Brazil; ^8^ The Feinstein Institutes for Medical Research, Northwell Health, Manhasset, NY, United States

**Keywords:** acute lung injury, acute respiratory distress syndrome, pyridostigmine, cholinergic stimulation, inflammation

## Abstract

Acute respiratory distress syndrome (ARDS) is a critical illness complication that is associated with high mortality. ARDS is documented in severe cases of COVID-19. No effective pharmacological treatments for ARDS are currently available. Dysfunctional immune responses and pulmonary and systemic inflammation are characteristic features of ARDS pathogenesis. Recent advances in our understanding of the regulation of inflammation point to an important role of the vagus-nerve-mediated inflammatory reflex and neural cholinergic signaling. We examined whether pharmacological cholinergic activation using a clinically approved (for myasthenia gravis) cholinergic drug, the acetylcholinesterase inhibitor pyridostigmine alters pulmonary and systemic inflammation in mice with lipopolysaccharide (LPS)-induced ARDS. Male C57Bl/6 mice received one intratracheal instillation of LPS or were sham manipulated (control). Both groups were treated with either vehicle or pyridostigmine (1.5 mg/kg twice daily, 3 mg/day) administered by oral gavage starting at 1 h post-LPS and euthanized 24 h after LPS administration. Other groups were either sham manipulated or received LPS for 3 days and were treated with vehicle or pyridostigmine and euthanized at 72 h. Pyridostigmine treatment reduced the increased total number of cells and neutrophils in the bronchoalveolar lavage fluid (BALF) in mice with ARDS at 24 and 72 h. Pyridostigmine also reduced the number of macrophages and lymphocytes at 72 h. In addition, pyridostigmine suppressed the levels of TNF, IL-1β, IL-6, and IFN-γ in BALF and plasma at 24 and 72 h. However, this cholinergic agent did not significantly altered BALF and plasma levels of the anti-inflammatory cytokine IL-10. Neither LPS nor pyridostigmine affected BALF IFN-γ and IL-10 levels at 24 h post-LPS. In conclusion, treatments with the cholinergic agent pyridostigmine ameliorate pulmonary and systemic inflammatory responses in mice with endotoxin-induced ARDS. Considering that pyridostigmine is a clinically approved drug, these findings are of substantial interest for implementing pyridostigmine in therapeutic strategies for ARDS.

## Introduction

Acute respiratory distress syndrome (ARDS) is a life-threatening form of acute lung injury and respiratory failure that presents a challenging medical problem (Fan et al., 2018). Globally, before 2020, ARDS affected approximately 3 million patients annually, accounting for more than 10% of intensive care unit admissions (Bellani et al., 2016; Fan et al., 2018). ARDS develops because of various pulmonary complications. Very recently, COVID-19 has alarmingly added another insult that may result in ARDS; ARDS is a major reason for COVID-19 patients’ admissions to intensive care units, and respiratory failure from ARDS is a major cause of mortality in these patients (Rodriguez-Morales et al., 2020; Ruan et al., 2020). Mortality in ARDS is within the range of 35–46% (Bellani et al., 2016). Despite many years of active research efforts, pharmacological treatments for ARDS are very limited and its management is largely based on supportive care—lung-protective mechanical ventilation (Fan et al., 2018). ARDS develops because of acute hypoxemia and non-hydrostatic pulmonary edema mediated through epithelium, alveolar macrophages, and vascular endothelium alterations, and acute, diffuse, inflammatory lung injury (Han and Mallampalli, 2015; Fan et al., 2018). Activated alveolar macrophages play a major role in ARDS inflammation through the recruitment of circulating neutrophils and macrophages, perpetuating an increased inflammatory response with the release of pro-inflammatory cytokines (Han and Mallampalli, 2015). Exacerbated pro-inflammatory cytokine release from mononuclear cells in ARDS mediates systemic inflammation affecting the lungs and other organs (Meduri et al., 2009; Han and Mallampalli, 2015). Increased lung and plasma pro-inflammatory cytokines, including TNF and IL-6 are detected in ARDS patients (Buttenschoen et al., 2008; Meduri et al., 2009; Han and Mallampalli, 2015). Endotoxemia in ARDS also is an important source of pro-inflammatory cytokines (Buttenschoen et al., 2008).

Inflammation is controlled by neural mechanisms and active research during the last 20 years has identified a role for a physiological mechanism—the vagus nerve-based *inflammatory reflex* in this regulation (Tracey, 2002; Pavlov and Tracey, 2017). In the inflammatory reflex, efferent vagus nerve cholinergic signaling mediated through the alpha 7 nicotinic acetylcholine receptor (α7nAChR) expressed on macrophages suppresses the production of TNF and other pro-inflammatory cytokines (Wang et al., 2003; Wang et al., 2004). The inflammatory reflex can be activated using electrical vagus nerve stimulation (VNS) and α7nAChR agonists (de Jonge and Ulloa, 2007; Pavlov, 2021). These approaches suppress the release of pro-inflammatory cytokines and alleviate disease severity in pre-clinical settings of numerous inflammatory conditions (Pavlov and Tracey, 2015). VNS *via* an implanted bioelectronic device also ameliorates inflammation and improves the disease scores in patients with inflammatory bowel disease and rheumatoid arthritis (Bonaz et al., 2016; Koopman et al., 2016; Genovese et al., 2020). Stimulation of brain cholinergic signaling has also been linked to activation of vagus nerve anti-inflammatory activity in the inflammatory reflex (Pavlov et al., 2006; Pavlov et al., 2009; Ji et al., 2014; Rosas-Ballina et al., 2015). Centrally acting cholinergic drugs such as the acetylcholinesterase (AChE) inhibitor galantamine alleviate inflammation in preclinical settings of endotoxemia, inflammatory bowel disease, lupus, metabolic syndrome and other disorders (Pavlov et al., 2009; Ji et al., 2014; Pavlov and Tracey, 2015; Pham et al., 2018; Lehner et al., 2019; Metz and Pavlov, 2020). Recently, galantamine was also shown to alleviate the inflammatory state and oxidative stress, alongside beneficial metabolic effects in patients with the metabolic syndrome (Consolim-Colombo et al., 2017; Sangaleti et al., 2021). The anti-inflammatory efficacy of activating cholinergic signaling using peripherally acting AChE inhibitors has also been explored (Pavlov and Tracey, 2015). However, this approach has generated mixed results with some evidence for efficacy (Miceli and Jacobson, 2003; Antunes et al., 2020), but also with negative results (Akinci et al., 2005; Kox et al., 2011) depending on the disease context.

Despite its potential clinical translatability, the efficacy of pharmacological cholinergic activation in acute lung injury and ARDS remains understudied. Previous reports have demonstrated the anti-inflammatory and protective effects of the α7nAChR agonists in murine models of mechanical ventilation-induced acute lung injury (Kox et al., 2011) hyperoxia-induced acute lung injury (Sitapara et al., 2020), radiation induced lung injury (Mei et al., 2018), and LPS-induced acute lung injury (Zhao et al., 2019). However, cholinergic activation using AChE inhibitors with relevance to future clinical translation in this context remains to be therapeutically explored. Pyridostigmine is a reversible AChE inhibitor and a cholinergic drug in current clinical use for the treatment of myasthenia gravis (Machado-Alba et al., 2017). Pyridostigmine (1.5 mg/kg) has been previously utilized in murine models (Kant et al., 2001; Silva et al., 2021) and shown to cause a 50% decrease in blood acetylcholinesterase levels (Kant et al., 2001). Treatment with pyridostigmine (3 mg/kg per day) has been demonstrated to decrease blood AChE activity by 85%, with no changes in blood butyrylcholinesterase after 3 or 7 days of treatment (Bernatova et al., 2003). Recent studies have shown that cholinergic stimulation with pyridostigmine decreases the heart rate (HR) and increases HR variability and baroreflex sensitivity in rats (Soares et al., 2004). These results concur with multiple studies suggesting that pyridostigmine may induce a protective effect against cardiovascular diseases (CVD) by both improving the vagal activity (de La Fuente et al., 2013; Lataro et al., 2013; Durand et al., 2014) and decreasing inflammatory responses (Feriani et al., 2017). Pyridostigmine treatment also improves survival, and pulmonary vascular remodeling in rats with pulmonary hypertension (da Silva Gonçalves Bós et al., 2018). While these previous studies with pyridostigmine focused on its cardiovascular effects, its potential to control pulmonary and systemic inflammation in ARDS remained unknown. Here we show that pyridostigmine administration significantly alleviates inflammation in a murine model of a direct LPS-induced acute lung injury and ARDS.

## Materials and Methods

### Chemicals and Reagents

Lipopolysaccharide (LPS, endotoxin, *E. Coli* 026:B6) and pyridostigmine (Pyridostigmine bromide) were purchased from Sigma-Aldrich^®^ (Saint Louis, MO) and dissolved in sterile, pyrogen-free PBS (Gibco^®^, Life Technologies, Grand Island, NY). Ketamine was purchased from Henry Schein animal health (Dublin, OH); and xylazine from Akron animal health (Lake Forest, IL, United States).

### Animals and Experimental Design

All experimental procedures were approved by the Institutional Animal Care and Use Committee of the Nove de Julho University (UNINOVE, AN0014/2015) and adhered to *The Guide for the Care and Use of Laboratory Animals* by the National Academy of Sciences as published by the National Institutes of Health (Copyright ^©^ 1996 by the National Academy of Sciences). Male 7–8 week-old C57Bl/6 mice (20–25 g) were obtained from the Animal House from Nove de Julho University, São Paulo, Brazil. Mice were maintained at temperature 21 ± 2°C, air humidity 50–60%, 12 h light/dark cycle, with free access to food and water (*ad libitum*). The effects of pyridostigmine treatments were investigated at 24 or 72 h after the onset of ARDS utilizing the following experimental design. Cohorts of anesthetized (ketamine 100 mg/kg and xylazine 10 mg/kg) mice were either sham manipulated (control group) or received one intratracheal instillation of 10 μg LPS per mouse to induce acute lung injury and ARDS. Both groups were treated with either vehicle (PBS) or pyridostigmine (1.5 mg/kg twice daily, 3 mg/day) administered by oral gavage starting at 1 h post-LPS. Mice were euthanized through exsanguinations at 24 h after the onset of ARDS. Other cohorts of mice were either sham manipulated (control group) or received 10 μg LPS/day/mouse for 3 days and were treated with vehicle or pyridostigmine, and euthanized at 72 h.

### Lipopolysaccharide Intratracheal Instilation

Mice were anesthetized (ketamine 100 mg/kg and xylazine 10 mg/kg) and received one intratracheal instillation of 10 μg per mouse of *Escherichia coli* LPS (026:B6; L3755, Sigma Aldrich, St. Louis, MO, United States) suspended in phosphate-buffered saline (PBS) to induce acute lung injury and ARDS. Briefly, anesthetized mice were positioned on an inclined platform (approximately 60–70°) with fixation at the incisors to gain free excess to the oral cavity and were hold the tongue with forceps to straighten the throat for instilling LPS into the trachea (proximal to the bifurcation) using a pipette (the injected volume depended on mouse bodyweight). The upper body of the mouse was kept in an upright position for 30 s to avoid leakage of the fluid from the trachea. The control groups received sterile PBS intratracheally instead of LPS. Once the mice recovered from anesthesia, they were returned into an individually ventilated cage and allowed free access to food and water.

### Blood Collection and Processing

Under anesthesia, 1 ml of blood was collected *via* cava vein and immediately centrifuged for 7 min at 950 *g* and 4°C. The plasma was collected and stored at—80°C for cytokine analyses.

### Cell Counts in Bronchoalveolar Lavage Fluid (BALF)

Following blood collection, the mice were euthanized by exsanguination, and the BALF was collected by flushing the lungs three times with 0.5 ml of 37°C sterile, pyrogen-free, physiological saline (0.9% NaCl) *via* the tracheal cannula. After the BALF collection, the samples were centrifuged for 10 min at 900 *g* and 4°C, and the supernatants were stored at −80°C for analysis. The cell pellets were resuspended in 1 ml of phosphate-buffered saline (PBS), and total cell counts were performed using a Neubauer chamber. Differential cell counts were performed using cytospin slides and stained with Diff-Quick. 300 cells were counted per slide.

### Bronchoalveolar Lavage Fluid and Plasma Cytokines Analyses

Cytokines were analyzed both in plasma and BALF. The levels of pro-inflammatory cytokines, including TNF-α, IL-1β, IL-6, IFN-γ and the anti-inflammatory cytokine IL-10 were analyzed by Enzyme-Linked Immunosorbent Assay (ELISA) with the respective kits from Biolegend (San Diego, CA) according to the manufacturer’s instructions. The SpectraMax I3 (Molecular Devices, San Diego, CA) was used to read the ELISA plates.

### Histomorphometric Analyses

The effects of pyridostigmine on parenchymal inflammation, a hallmark of ARDS, were analyzed by quantitative histomorphometric image analysis. Lungs were collected and fixed in 10% formalin and processed. Briefly, 5 μm thickness lung slices were stained with hematoxylin and eosin. 15 random fields of the lung parenchyma of each mouse were photographed. The air and tissue area of all photomicrographs were determined using the software Image Pro Plus 4.5 (MediaCybernetics, Silver Spring, MD). The number of neutrophils was counted in each photo according to the morphological criteria by an experienced investigator, blinded to the experimental group’s description. Then, the number of neutrophils was presented as per square millimeter of lung tissue (Rigonato-Oliveira et al., 2018).

### Statistical Analysis

Data analysis was performed using GraphPad Prism (GraphPad Software, La Jolla, California). Values are presented as mean ± the standard error of the mean (SEM). For parametric data, the one-way analysis of variance (one-way ANOVA) was performed with Turkey’s multiple comparison tests. For nonparametric data, the Kruskal-Wallis test was used. *p* values < 0.05 were considered statistically significant.

## Results

### Pyridostigmine Reduced Transmigration of Neutrophils During Acute Respiratory Distress Syndrome

**FIGURE 1:**
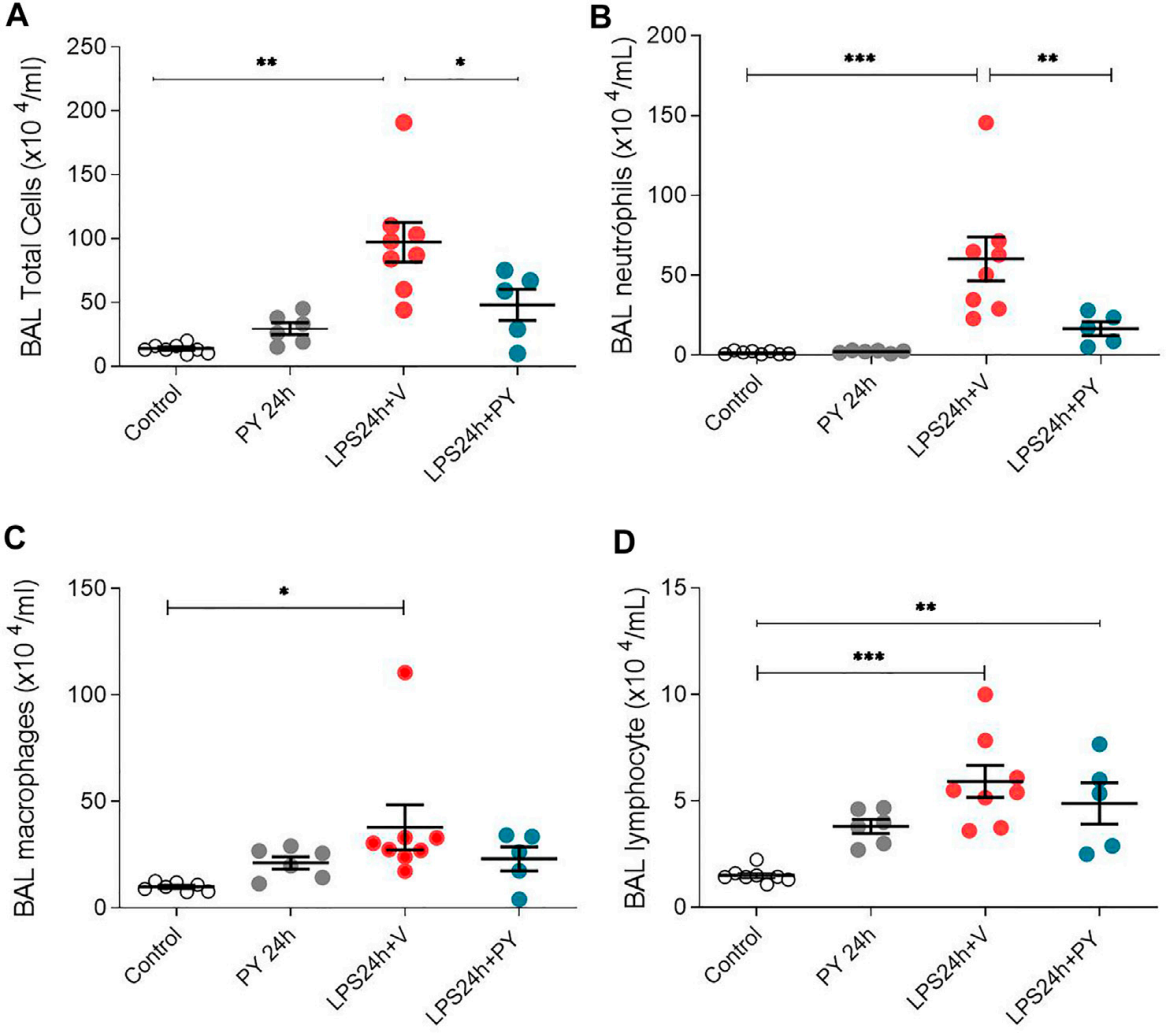
Pyridostigmine reduces BAL immune/inflammatory cell numbers in LPS exposed mice at 24 h. **(A)** BAL total cells **(B)** neutrophils, **(C)** macrophages, and **(D)** lymphocytes. Vehicle (V) group (Control), pyiridostigmine (PY), lipopolysaccharide (LPS) ***p < 0.001, **p < 0.01 and *p < 0.05. Values expressed as mean ± SEM. One-way ANOVA followed by Tukey’s test (See text for details).

**FIGURE 2:**
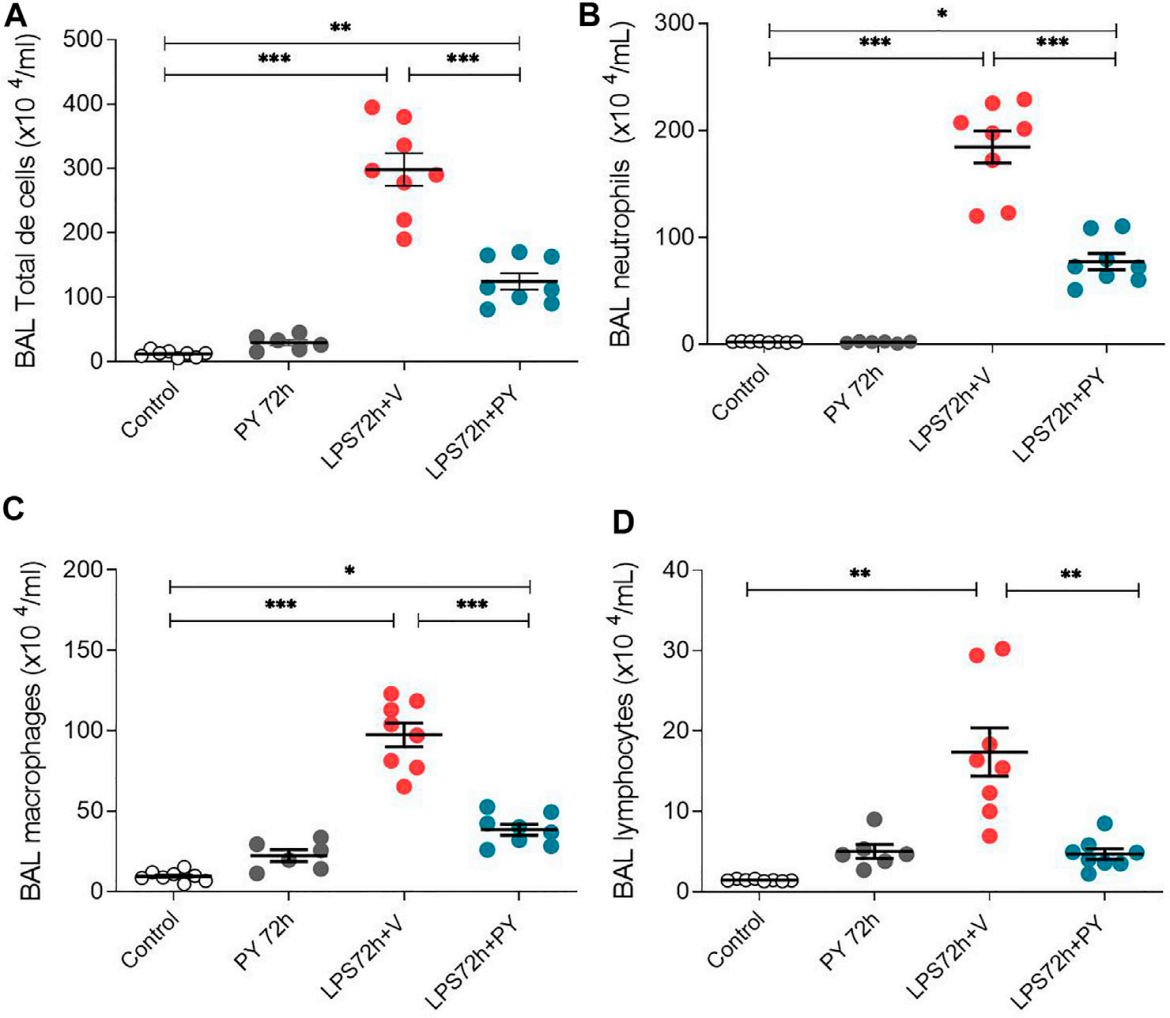
Pyridostigmine reduces BAL immune/inflammatory cell numbers in LPS exposed mice at 72 h. **(A)** BAL total cells **(B)** neutrophils, **(C)** macrophages, and **(D)** lymphocytes. Vehicle (V) group (Control), pyiridostigmine (PY), lipopolysaccharide (LPS) ***p < 0.001, **p < 0.01 and *p < 0.05. Values expressed as mean ± SEM. One-way ANOVA followed by Tukey’s test (See text for details).

**FIGURE 3:**
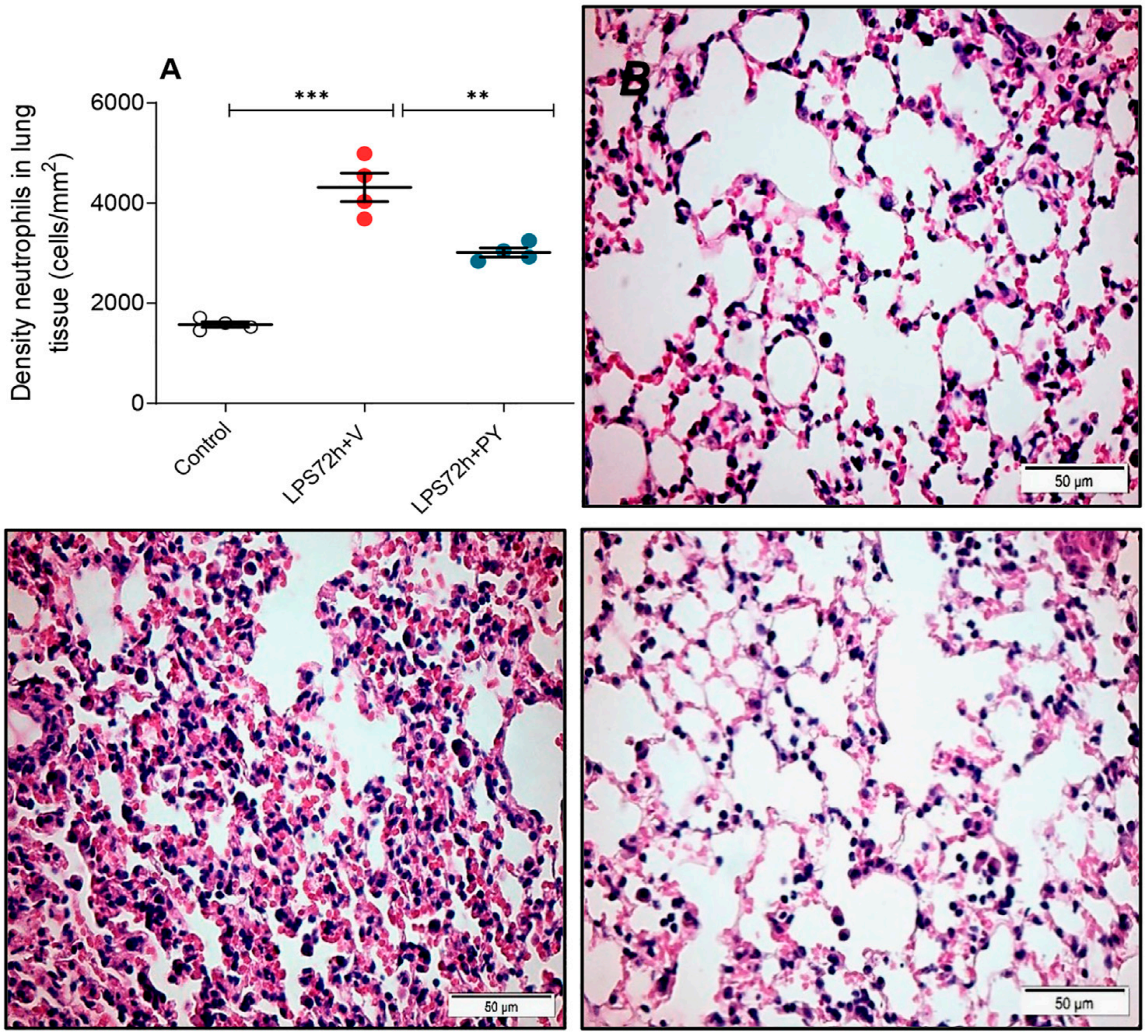
Pyridostigmine decreases the density of neutrophils in lung tissue of LPS exposed mice at 72 h. **(A)** Quantification of neutrophils ***p < 0.00, **p < 0.01. **(B, C, D)** Representative photomicrographs of lung tissues stained with hematoxylin-eosin **(B)** control **(C)** LPS + vehicle **(D)** LPS + pyridostigmine. Magnification, ×400. Values expressed as mean ± SEM. One-way ANOVA followed by Tukey’s test (See text for details).

**FIGURE 4:**
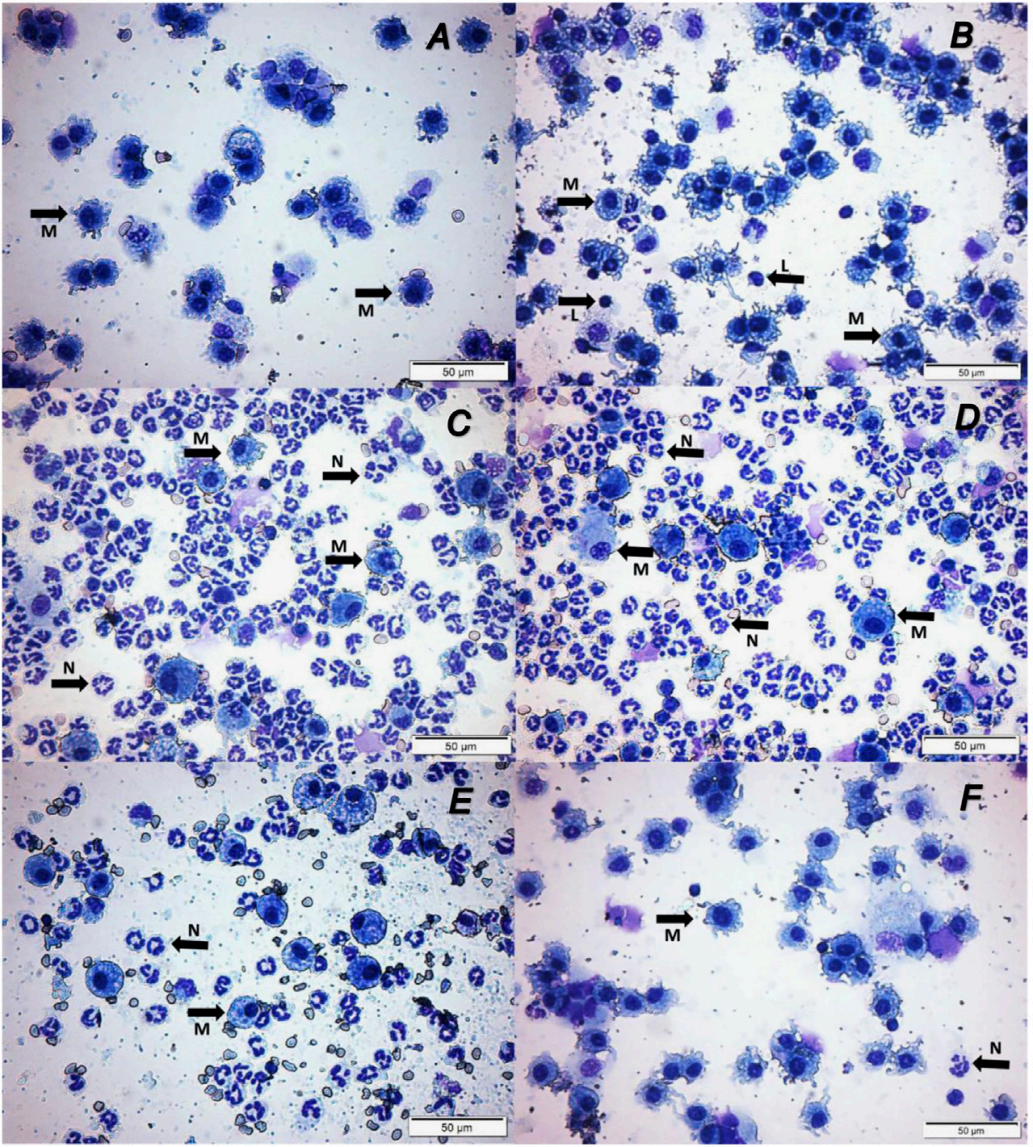
Pyridostigmine treatment results in lower levels of numbers of macrophages, neutrophils, and lymphocytes in BAL **(**Representative photomicrographs differential cells in BAL). **(A)** Control at 24 h, **(B)** Control at 72 h, **(C)** LPS + vehicle at 24 h, **(D)** LPS + vehicle at 72 h, **(E)** LPS + pyridostigmine at 24 h, and **(F)** LPS + pyridostigmine at 72 h. The arrows in the images indicate some of the cells with morphology of neutrophils and macrophages (See the text for details).

We examined whether enhancing cholinergic signaling by pyridostigmine affected the cellular responses during LPS-induced acute lung injury and ARDS by first analyzing the number of inflammatory cells (total and differential cell count) in the BALF at 24 and 72 h. Pyridostigmine administration did not significantly alter either of the parameters studied in the control group (Figure 1). Endotoxin administration significantly increased the number of total cells, neutrophils, macrophages, and lymphocytes (Figures 1A–D) in the BALF compared with control animals at 24 h. Treatment with pyridostigmine of mice with ARDS (compared with vehicle) significantly reduced the number of both total cells and neutrophils (Figures 1A,B). However, treatment with pyridostigmine of mice with ARDS did not have any significant effect on macrophages and increased the number of lymphocytes (compared with controls) at 24 h. At 72 h pyridostigmine administration did not alter BALF cell numbers compared with control (Figure 2). At the same time point (72 h) intratracheal LPS-challenge (compared with control) significantly increased the number of total cells (Figure 2A), neutrophils (Figure 2B), macrophages (Figure 2C), and lymphocytes (Figure 2D) in the BALF. Treatment with pyridostigmine of mice with ARDS (compared with vehicle) significantly reduced the number of total cells, neutrophils, macrophages, and lymphocytes (Figures 2A–D). Likewise, histological examination and quantification revealed that pyridostimine treatment (compared with vehicle) significantly reduced the number of neutrophils in the lung tissue of ARDS mice at 72 h (Figure 3A). Histological analysis also indicated that pyridostigmine reduced the neutrophil numbers in the BALF of ARDS mice at 24 and 72 h (Figure 4). Together these results indicate that treatment with pyridostigmine reduces the migration and accumulation of immune/inflammatory cells into the lung at 24 and 72 h during ARDS in mice.

### Pyridostigmine Reduced Pro-inflammatory Cytokine Levels During Intratracheal Lipopolysaccharide-induced Acute Respiratory Distress Syndrome

**FIGURE 5:**
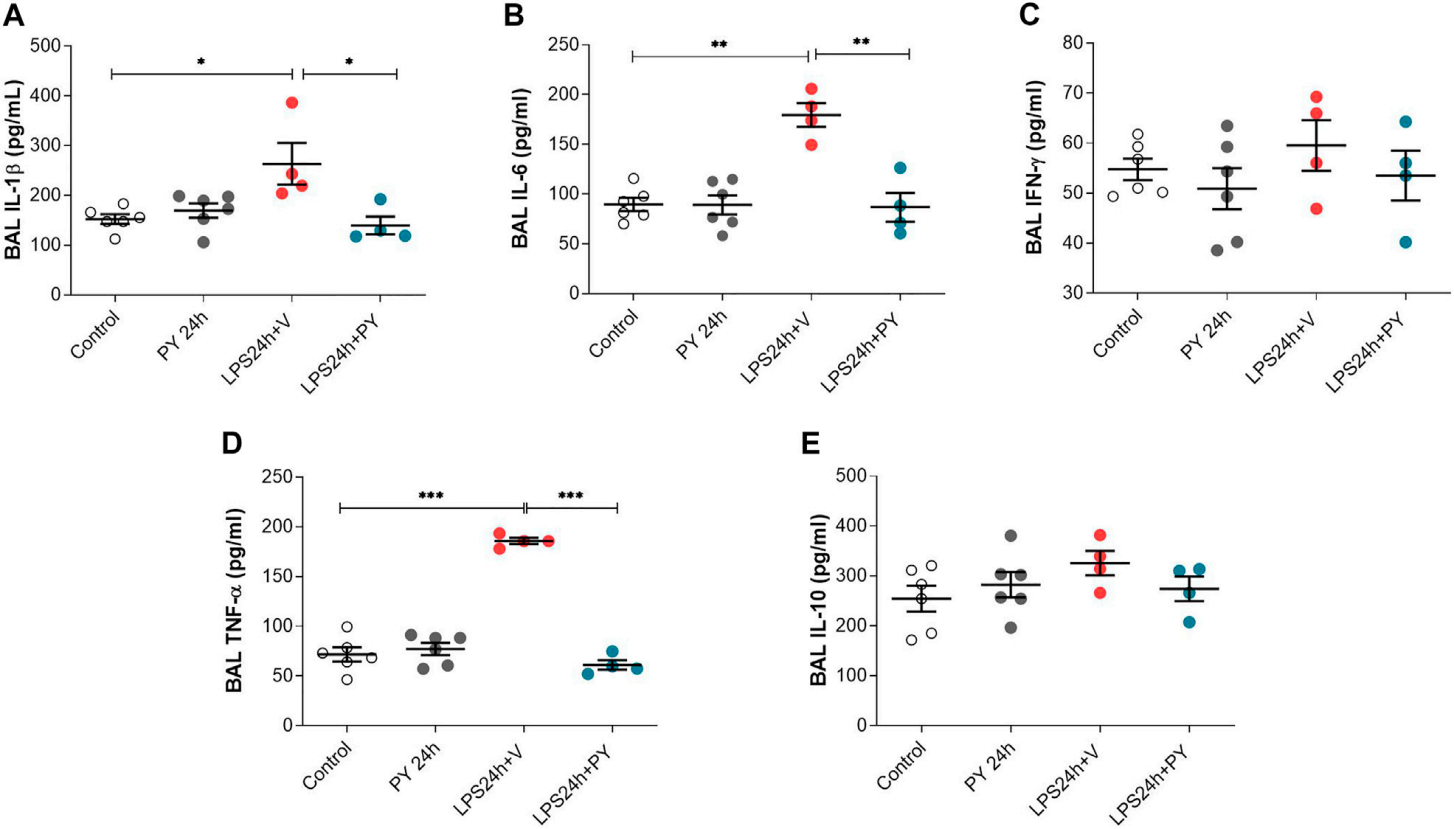
Physostigmine decreases pro-inflammatory cytokine levels in BAL at 24 h. **(A)** IL-1β, **(B)** IL-6, **(C)** IFN-γ, **(D)** TNF-α, **(E)** IL-10. ***p < 0.001, **p < 0.01 and *p < 0.05. Vehicle (V) group (Control), pyiridostigmine (PY), lipopolysaccharide (LPS). Values expressed as mean ± SEM. One-way ANOVA followed by Tukey’s test (See text for details).

**FIGURE 6:**
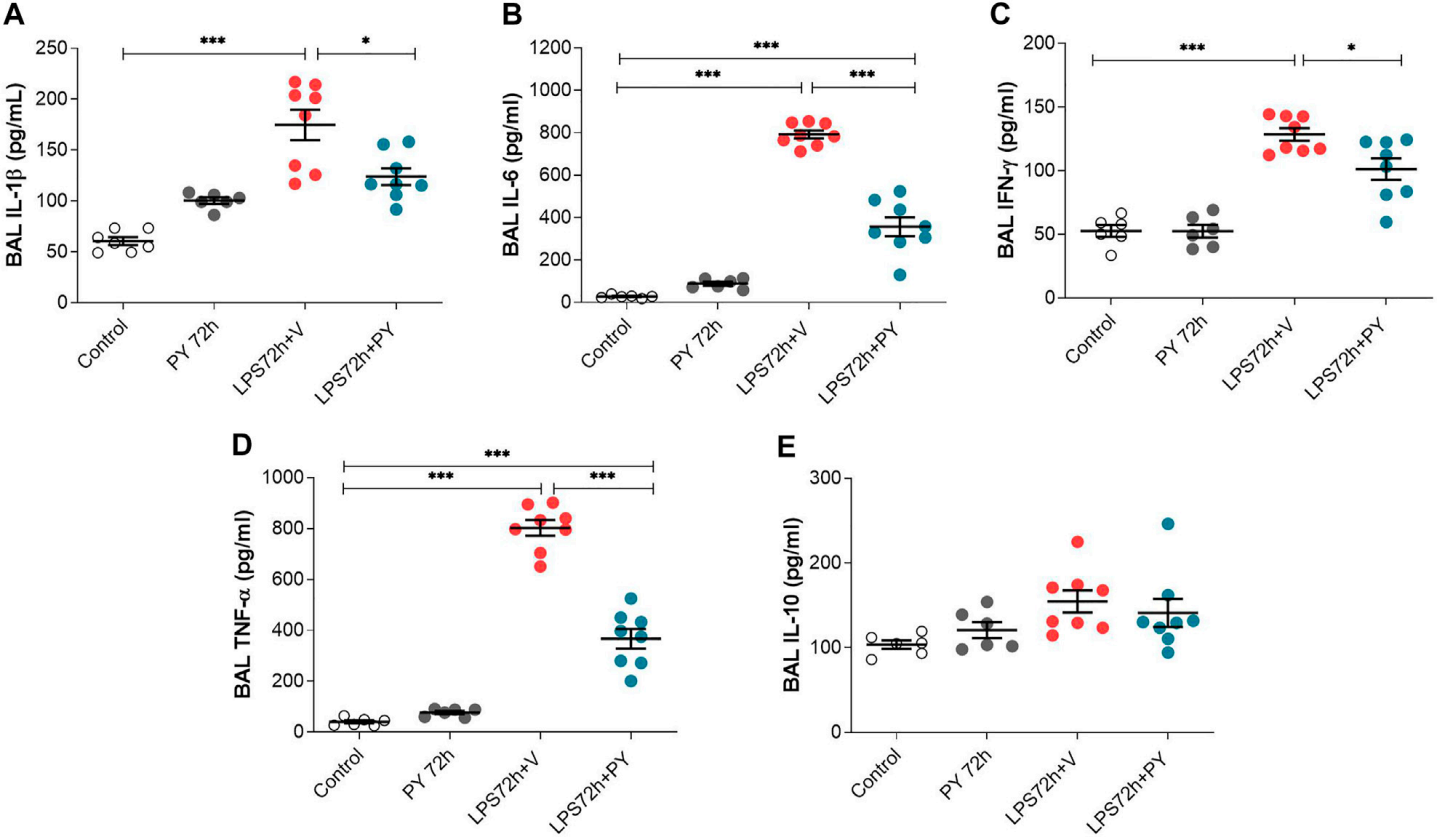
Pyridostigmine suppresses BAL pro-inflammatory cytokine levels in LPS exposed mice at 72 h. **(A)** IL-1β **(B)** IL-6, **(C)** IFN-γ, **(D)** TNF-α, **(E)** IL-10. ***p < 0.001 and *p < 0.05. Vehicle (V) group (Control), pyiridostigmine (PY), lipopolysaccharide (LPS). Values expressed as mean ± SEM. One-way ANOVA followed by Tukey’s test (See text for details).

We next examined the effects of piridostigmine on the molecular immune responses in LPS-induced ARDS by analyzing BALF levels of cytokines, including TNF, IL-1β, IL-6, and INF-γ, and IL-10 at 24 and 72 h. Treatment with pyridostigmine (compared with controls) did not significantly affect any of the cytokines analyzed in the BALF at 24 h (Figure 5). LPS significantly increased the levels of the pro-inflammatory cytokines IL-1β (Figure 5A), IL-6 (Figure 5B), and TNF (Figure 5D), but did not affect the levels of INF-γ (Figure 5C) in the BALF of the mice at 24 h. Pyridostigmine treatment of mice with ARDS significantly decreased the levels of IL-1β (Figure 5A), IL-6 (Figure 5B), and TNF (Figure 5D). The effects of pyridostigmine were specific for those cytokines induced by intratracheal LPS. LPS (compared with control) did not significantly alter INF-γ (Figure 5C) in the BALF and pyridostigmine treatment had no significant effects. Likewise, neither LPS nor treatment with pyridostigmine affected the levels of the anti-inflammatory cytokine IL-10 in the BALF at 24 h (Figure 5E). Pyridostigmine treatment (compared with control) did not significantly alter cytokines analyzed in the BALF at 72 h post-treatment (Figure 6). Intratracheal LPS significantly increased the levels of IL-1β (Figure 6A), IL-6 (Figure 6B), INF-γ (Figure 6C), and TNF (Figure 6D) in the BALF at 72 h. However, LPS administration did not significantly alter BALF levels of the anti-inflammatory cytokine IL-10 (Figure 6E). Pyridostigmine treatment (compared with vehicle) significantly lowered IL-1β (Figure 6A), IL-6 (Figure 6B), INF-γ (Figure 6C), and TNF (Figure 6D) levels in the ARDS mice. Similar to that reported at 24 h, neither LPS nor treatment with pyridostigmine affected the levels anti-inflammatory cytokine IL-10 in the BALF at 72 h (Figure 6E). Of note, while there were significant increases in BALF IL-6, INF-γ, and TNF levels (*p* < 0.001) at 72 h compared with 24 h, IL-10 levels were lower (*p* < 0.001) and IL-1β levels were not significantly altered (Figures 5, 6). Together, these results indicate that pyridostigmine treatment induces significant anti-inflammatory effects in the lungs of ARDS mice at 24 and 72 h and does not merely delay the generation of inflammatory responses.

### Pyridostigmine Reduced Systemic Inflammation in Mice With Acute Respiratory Distress Syndrome

**FIGURE 7:**
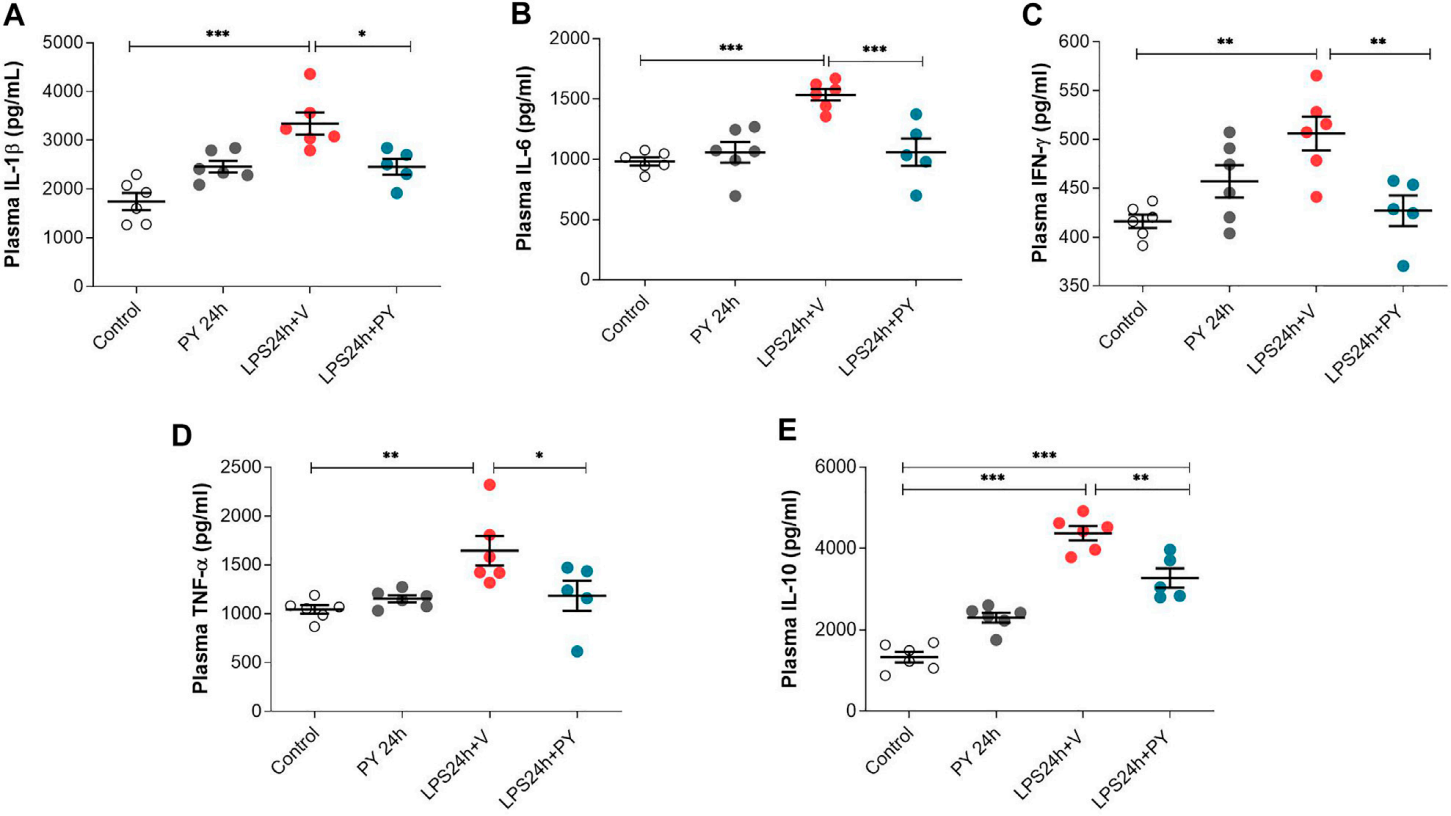
Pyridostigmine suppresses plasma cytokine levels in LPS exposed mice at 24 h. **(A)** IL-1β, **(B)** IL-6, **(C)** IFN-γ, **(D)** TNF-α, **(E)** IL-10. ***p < 0.001, **p < 0.01 and *p < 0.05. Vehicle (V) group (Control), pyiridostigmine (PY), lipopolysaccharide (LPS). Values expressed as mean ± SEM. One-way ANOVA followed by Tukey’s test (See text for details).

**FIGURE 8:**
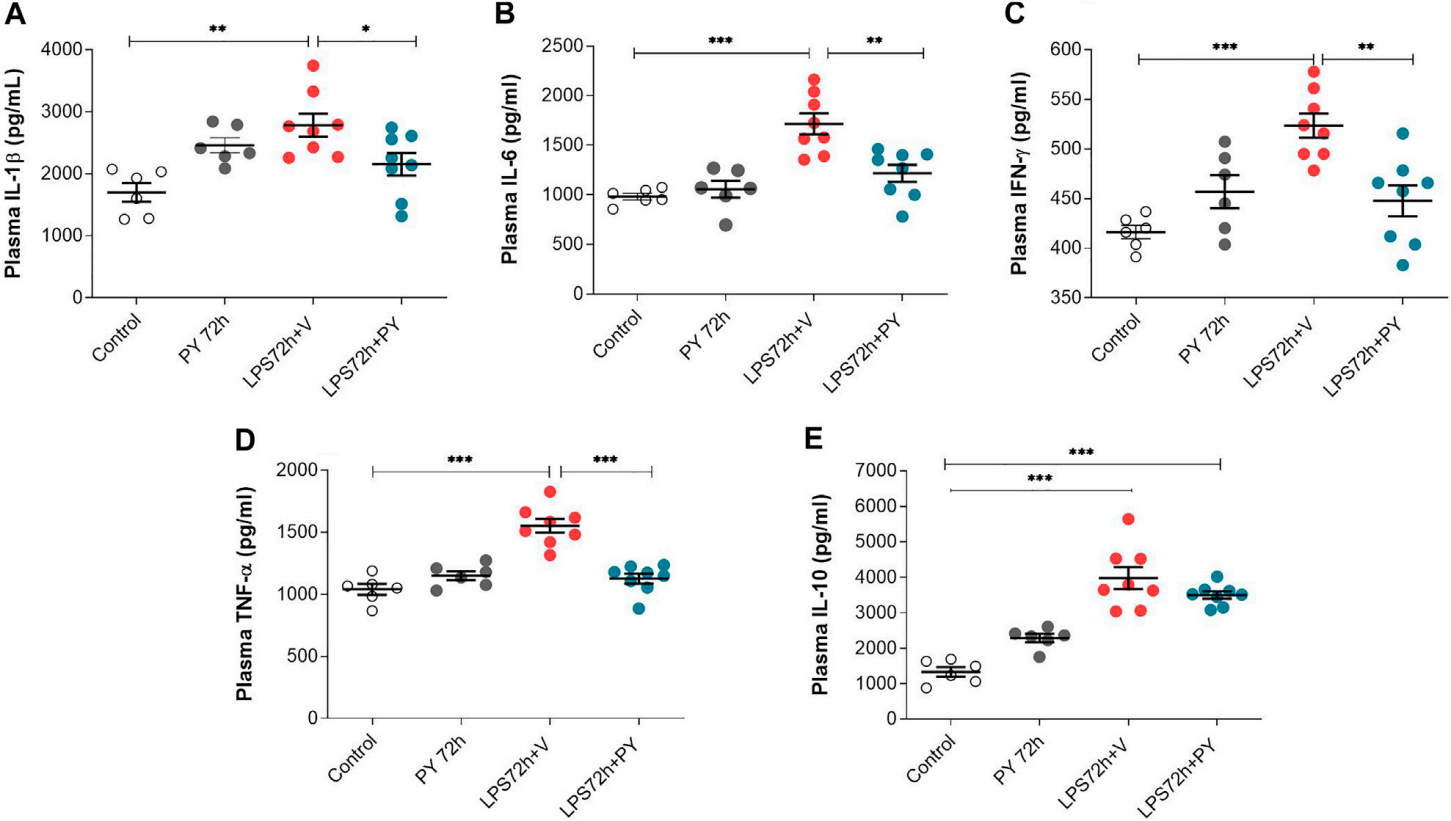
Pyridostigmine suppresses plasma cytokine levels in LPS exposed mice at 72 h. **(A)** IL-1β, **(B)** IL-6, **(C)** IFN-γ, **(D)** TNF-α, **(E)** IL-10. ***p < 0.001, **p < 0.01 and *p < 0.05. Vehicle (V) group (Control), pyridostigmine (PY), lipopolysaccharide (LPS). Values expressed as mean ± SEM. One-way ANOVA followed by Tukey’s test (See text for details).

The effects of pyridostigmine on innate immune responses and systemic inflammation during LPS-induced ARDS were studied by analyzing plasma cytokine levels at 24 and 72 h. Treatment with pyridostigmine (compared with control) did not affect any of the plasma cytokine levels (Figure 7). Intratracheal LPS significantly increased plasma levels of IL-1β (Figure 7A), IL-6 (Figure 7B), INF-γ (Figure 7C), and TNF (Figure 7D) at 24 h. LPS also increased the plasma levels of the anti-inflammatory cytokine IL-10 (Figure 7E). Treatment with pyridostigmine significantly suppressed plasma IL-1β (Figure 7A), IL-6 (Figure 7B), INF-γ (Figure 7C), TNF (Figure 7D), and IL-10 (Figure 7E) levels in mice with LPS-induced ARDS at 24 h. At 72 h pyridostigmine (compared with control) did not affect any of the plasma cytokines analyzed (Figure 8). LPS administration resulted in a significant systemic inflammatory response manifested by increased plasma levels of all cytokines measured, including IL-1β (Figure 8A), IL-6 (Figure 8B), INF-γ (Figure 8C), TNF (Figure 8D), and IL-10 (Figure 8E) at 72 h. Treatment with pyridostigmine started after endotoxin administration significantly lowered IL-1β (Figure 8A), IL-6 (Figure 8B), INF-γ (Figure 8C), and TNF (Figure 8D). However, the drug treatment failed to significantly alter plasma levels of the anti-inflammatory cytokine IL-10 (Figure 8E). These results demonstrate the systemic anti-inflammatory effects of pyridostigmine in mice with ARDS.

## Discussion

Here we report that the AChE inhibitor and a cholinergic drug pyridostigmine attenuates inflammation in mice with LPS-induced acute lung injury and ARDS. Our results demonstrate that pyridostigmine treatments significantly reduce the number of neutrophils and total immune/inflammatory cells. Importantly, pyridostigmine administration was initiated at 1 h after the LPS challenge—a time point, which is reportedly after neutrophil infiltration into the lung has started (Rigonato-Oliveira et al., 2018). In addition to the effectiveness of pyridostigmine in reducing these cellular components of the inflammatory response, the results also demonstrate that pyridostigmine significantly suppresses the systemic pro-inflammatory state during experimental ARDS.

As previously reported, intratracheal LPS-induced ARDS is characterized by initial increase of polymorphonuclear (PMN) cells, albumin, and cytokines (including TNF, IL-1β, IL-6, IL-8, MCP-1, MIP-1) in the BALF, and then (between 24 and 48 h after instillation) there is an additional increase in PMN, monocytes, macrophages, and lymphocytes (Matute-Bello et al., 2008). In our experimental model, we observed a marked pulmonary inflammatory response at 24 or 72 h after the intratracheal LPS challenge. Acute lung injury was indicated by an increase of neutrophils in the lung tissue and by an increase of the number of neutrophils and macrophages in the BALF. This was followed by an increase in the levels of pro-inflammatory (TNF, IL-1β, IL-6) and the anti-inflammatory cytokine IL-10. In this context, treatment with pyridostigmine started after the intratracheal LPS-challenge suppressed neutrophil migration and accumulation into the lungs, as shown by the reduced neutrophil counts in the BALF and the quantitative analysis of neutrophils density in the lung parenchyma. Pyridostigmine also reduced the number of total cells, neutrophils, and macrophages in the BALF, and neutrophil density in the lung tissue at 72 h.

The increased release of cytokines, including TNF, IL-1β, IL-6, INF-γ, and IL-8, from activated macrophages and other cells importantly mediates and exacerbates the pathophysiology of ARDS (Johnston et al., 2012). TNF is a critical inflammatory molecule involved in the adhesion and activation of neutrophils, as well as the coagulation and edema formation especially during acute pulmonary inflammation (Aimbire et al., 2006; Johnston et al., 2012). TNF amplifies the inflammatory response by stimulating the production of IL-6, which plays a central role in the inflammatory process in ARDS (Aimbire et al., 2006; Johnston et al., 2012; Spadaro et al., 2019). Pyridostigmine significantly decreased TNF levels in BALF and plasma at 24 and 72 h. Pyridostigmine also significantly lowered IL-6 both in the BALF and plasma at both time points. Another cytokine that is increased in the BALF of ARDS patients and correlated with poor prognosis is IL-1β (Meduri et al., 1995). Treatment with pyridostigmine significantly reduced IL-1β levels both in BALF and plasma at both time points. IFN-γ is a Th1-associated cytokine with a key mediating role in the influx of leukocytes into the lungs ARDS and a proposed marker of ARDS (Spadaro et al., 2019). Our results indicate a time-dependent effect of intratracheal LPS on plasma and BALF levels; at 24 h these levels were increased in plasma only while both plasma and BALF levels were increased at 72 h. Pyridostigmine treatment effectively suppressed LPS-induced plasma and BALF IFN-γ levels. Of note, the higher BALF levels of IL-6, IFN-γ and TNF at 72 h (compared with 24 h) indicated a trend toward exacerbation of cytokine-mediated local inflammatory responses at the later time point, which is expected considering the continued LPS administration. However, no further exacerbation of systemic inflammation was detected, because plasma cytokine levels remained similar. These observations suggest time-dependent differential alterations in the lung and systemic inflammatory responses.

Pyridostigmine is an AChE inhibitor, which in the doses used in this study significantly decreases AChE activity (Kant et al., 2001; Bernatova et al., 2003). Although not established at this point, activation of cholinergic signaling in the efferent arm of the inflammatory reflex by pyridostigmine can be suggested as a plausible mechanism underlying pyridostigmine suppression of pro-inflammatory cytokine levels. This line of reasoning is supported by the documented involvement of cholinergic signaling through α7nAChR-mediated mechanisms in the inflammatory reflex (Wang et al., 2003; de Jonge and Ulloa, 2007) and the anti-inflammatory therapeutic efficacy of VNS and α7nAChR agonists in models of acute lung injury (Su et al., 2007; dos Santos et al., 2011; Sitapara et al., 2020). Another observation from our study with relevance to the inflammatory reflex is the lack of effect of pyridostigmine on the anti-inflammatory cytokine IL-10. Previous findings have demonstrated that while VNS, which activates the inflammatory reflex and its efferent cholinergic arm, results in suppression of pro-inflammatory cytokine levels, this approach does not have an effect of IL-10 levels (Borovikova et al., 2000; Huston et al., 2006; Rosas-Ballina et al., 2011; Olofsson et al., 2017). At early stages of acute lung injury and ARDS, alveolar macrophages initiate pro-inflammatory signaling through the release of TNF, IL-1β and other cytokines that propagates the innate immune response and inflammation and potentiates chemokine secretion to recruit neutrophils, exudative macrophages, and lymphocytes (Herold et al., 2011; Aggarwal et al., 2014). Although the mechanisms of pyridostigmine effects related to reducing immune cell migration and accumulation in the lungs remain to be elucidated, activation of cholinergic signaling that results in reduced pro-inflammatory cytokine release can be suggested as a key mediating event.

Future studies should provide additional relevant insights into pyridostigmine effects on a broader scope of physiological indices, including the respiratory function (i.e. O2 consumption, and lung mechanics) in the context of acute lung injury and ARDS. The findings of this study also provide a rationale for future studies with pyridostigmine utilizing other murine models, including cecal ligation and puncture-induced polymicrobial sepsis in the paradigm of extrapulmonary indirect causes of acute lung injury and ARDS.

ARDS clinical management remains challenging and limited therapeutic options are available in addition to supportive care—lung-protective mechanical ventilation (Fan et al., 2018). Acute lung injury and ARDS also develop in severe cases of COVID-19 and the associated cytokine storm has been proposed as a therapeutic target (Andersson et al., 2020; Feldmann et al., 2020; Mehta et al., 2020). Cholinergic signaling within the vagus nerve-based inflammatory reflex is a key physiological regulator of cytokine release and inflammation (Tracey, 2002; Pavlov and Tracey, 2017; Pavlov, 2019). There is a growing interest in utilizing several approaches of non-invasive bioelectronic VNS and pharmacological activation of cholinergic signaling in the treatment of COVID-19 (Bonaz et al., 2020; Farsalinos et al., 2020; Staats et al.,2020; Tindle et al., 2020) manifested by a number of ongoing clinical trials. There is also an ongoing clinical trial with pyridostigmine with COVID-19 patients: NCT04343963.

Therefore, these findings are of significant interest for further developing pyridostigmine or other clinically approved AChE inhibitors in therapeutic strategies for ARDS, including in the context of COVID-19.

## Data Availability

The raw data supporting the conclusions of this article will be made available by the authors, without undue reservation.
